# Improve the Detection Range of Semi-Active Laser Guidance System by Temperature Compensation of Four-Quadrant PIN Detector

**DOI:** 10.3390/s19102284

**Published:** 2019-05-17

**Authors:** Siyuan Gao, Hui Liu, Hongwei Zhang, Xin Zhang, Juan Chen

**Affiliations:** 1 Changchun Institute of Optics, Fine Mechanics and Physics, Chinese Academy of Sciences, Changchun 130033, China; liuh6009@163.com (H.L.); zhanghw135@163.com (H.Z.); zhangxin@tju.edu.cn (X.Z.); stjuanzyh@gmail.com (J.C.); 2 University of Chinese Academy of Sciences, Beijing 100049, China

**Keywords:** quadrant PIN detector (QD), temperature compensation, responsivity, semi-active laser guidance

## Abstract

The detection range of a semi-active laser guidance system can deviate significantly from the design value over a wide ambient temperature range. In this paper, a mathematical model of the detection range of a semi-active laser guidance system is built and the main factors affecting the detection range are analyzed. The parameter responsivity, which shows significant change, is found by applying the ambient temperature stress to the four-quadrant PIN detector and its signal processing chain. The relationship between the maximum detection range and ambient temperature is established based on a given signal-to-noise ratio, which is necessary for reliable detection. The target temperature and tolerance are setup for real-time temperature compensation for the four-quadrant PIN detector. The ambient temperature stress is applied to the system under compensation to verify the effect of compensation. The experimental results show that the ratio of the maximum variation of the detection range to the design point is 6.9% after the compensation is implemented when the ambient temperature changes from −40 °C to 60 °C, which is improved by 13.2% compared to that without compensation.

## 1. Introduction

The four-quadrant detector (QD) is a position-sensitive device, which has higher sensitivity, excellent dynamic response and lower noise compared to lateral effect PSD and CCD. It can be used to measure small displacements [1]. Based on the above characteristics, a four-quadrant detector can be used for precision error measurement [2], optical tweezers [3], laser communication [4] and laser guidance [5].

Current research directions for four-quadrant detectors are generally divided into two categories. One is to study the physical properties of a four-quadrant detector, which influence measurement accuracy. The physical properties of a four-quadrant detector include noise, crosstalk and dead zone. There are many achievements in this field. Zhang et al. studied the noise characteristics of four-quadrant detectors [6]. Ma et al. analyzed the influence of crosstalk on the measurement accuracy of four-quadrant detectors [7]. Zhang et al. analyzed the influence of dead zone on the position measurement accuracy of four-quadrant detectors [8]. Gao et al. studied the influence of the different characteristics between the four channels of the four-quadrant detector and the post-processing circuit [9]. Lee et al. studied the influence of spot size on position detection sensitivity [10].

The other research direction is to study the position detection algorithm of four-quadrant detectors. Hao et al. studied the layout of the four-quadrant detector and pointed out that a 45° tilting arrangement has a higher sensitivity than the conventional method when the spot is near the center of the detector [11]. Lu et al. compared the effect of uniform distribution of energy density and Gaussian distribution of energy density on position detection accuracy [12]. Li et al. proposed a new method to improve the position measurement accuracy for Laguerre-Gaussian beams on a QD [13]. Chen et al. used a polynomial fitting method to solve the spot position on the QD [14]. Cui et al. improved measurement accuracy of the quadrant detector through improvement of the linearity index [15]. Wu et al. proposed a new formula to improve the accuracy of spot position on QDs by combining the infinite integral method with the Boltzmann method, due to their opposite error characteristics [16].

Most of the above studies were carried out under stable ambient temperature conditions, such as room temperature 25 °C. However, the ambient temperature of the four-quadrant PIN detector varies widely in the semi-active laser guidance system, between −40 °C to +60 °C. The parameters of the four-quadrant PIN detector and its signal processing chain can vary significantly within this temperature range. These parameters affect the maximum detection range in the semi-active laser guidance system directly. Therefore, it is necessary to measure and compensate the temperature characteristics of the four-quadrant PIN detector and its signal processing chain, so that the maximum detection range of the laser guidance system is kept within a reasonable and usable range over the entire ambient temperature range.

Inspired by the above researchers, the contents of this paper are as follows. The second part of the paper sets up the mathematical model of the detection range in the semi-active laser guidance system and analyzes the main factors affecting the detection range. The third part sets up observation points in the analog signal chain, monitors each point within the range of temperature variation, and fits the observation data to obtain the temperature variation characteristics of each main parameter in the model. The fourth part picks out the parameters with significantly different temperature characteristics and performs temperature compensation. In the fifth part, the temperature stress is applied to the compensated system, and the maximum detection range of compensated system is compared to the system which is not compensated. The validity of the proposed compensation method is verified.

## 2. Mathematical Model of Detection Range in Semi-Active Laser Guidance System

**Figure 1:**
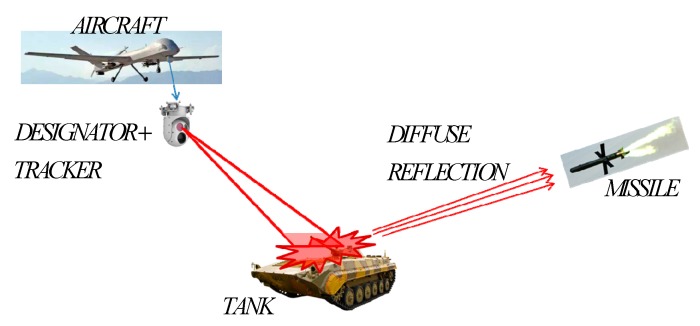
Schematic diagram of the laser semi-active guidance system.

The typical working mode of the semi-active laser guidance system is shown in Figure 1. The designator and the image tracking system with visible or infrared band are mounted on a gyro-stabilized platform which is loaded onto the aircraft as a payload. The designator keeps the spot illuminating on the target object with assistance of the image tracker. The seeker of the semi-active laser guidance system can detect the echo signals of the illuminated target in real time, within an effective range and calculate the angle of sight line which can generate guidance information to guide a missile to the target.

**Figure 2:**
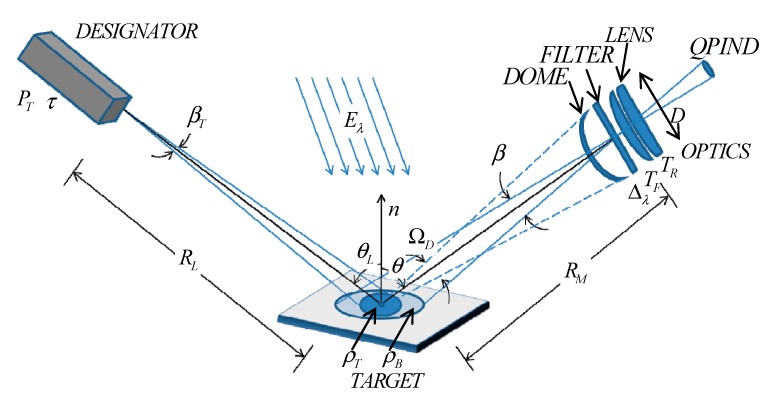
Modeling of semi-active laser guidance system.

The parts in Figure 1 are abstracted and modeled, as shown in Figure 2.

$R_{L}$ is the distance between the laser designator and the target. The laser pulse emitted by the designator is approximately Gaussian. The full-width at half-maximum (FWHM) of the laser pulse equals to τ. $E_{T}$ is the energy of pulse, $\beta_{T}$ is the laser beam angle, σ is the attenuation coefficient of laser in atmosphere, $\theta_{L}$ is the angle between the object surface normal and the laser beam. Considering the background and the object, a diffuse reflector is assumed, which is reflected as a Lambertian source [17]. $\rho_{T}$ is the target reflectance.

Therefore, the peak power of the laser pulse of designator can be calculated as:

(1) $$P_{L}=\frac{E_{T}}{\tau }$$

The spectral radiance is:

(2) $$L_{T}=\frac{4P_{L}\rho_{T}e^{-\sigma R_{L}}\cos\theta_{L}}{\pi \beta_{T}^{2}R_{L}^{2}}$$

Assuming that the laser spot falls on the target totally, the area of laser spot on the target is:

(3) $$A_{T}=\frac{\pi \beta_{T}^{2}R_{L}^{2}}{4\cos\theta_{L}}$$

And the solid angle of the spot to the optics is:

(4) $$\Omega_{D}\approx \frac{\pi D^{2}}{4R_{M}^{2}}$$

D is the optical aperture of the optical system, $R_{M}$ is the distance between the target and the receiving optics. The received optical signal power, $P_{S}$, of the reflected laser radiation from the illuminated object is [18]:

(5) $$P_{S}=L_{T}A_{T}\Omega_{D}T_{R}T_{F}e^{-\sigma \left(R_{M}+R_{L}\right)}\cos\theta$$

$T_{R}$ is the receiving optics transmission coefficient, $T_{F}$ is the optical filter transmission coefficient. Combing Equations (1)–(4) with Equation (5) we can obtain the $P_{S}$ as follows:

(6) $$P_{S}=\frac{D^{2}}{4R_{M}^{2}}P_{L}\rho_{T}T_{R}T_{F}e^{-\sigma \left(R_{M}+R_{L}\right)}\cos\theta$$

The received optical signal power of the reflected background radiation from the solar is:

(7) $$P_{B}=\frac{\pi }{16}E_{\lambda }\Delta_{\lambda }\rho_{B}\beta D^{2}T_{R}T_{F}e^{-\sigma R_{M}}\cos\theta$$

where $E_{\lambda }$ is the solar spectral irradiance, $\Delta_{\lambda }$ is the optical spectral filter bandwidth and $\rho_{B}$ is the background reflectance.

**Figure 3:**
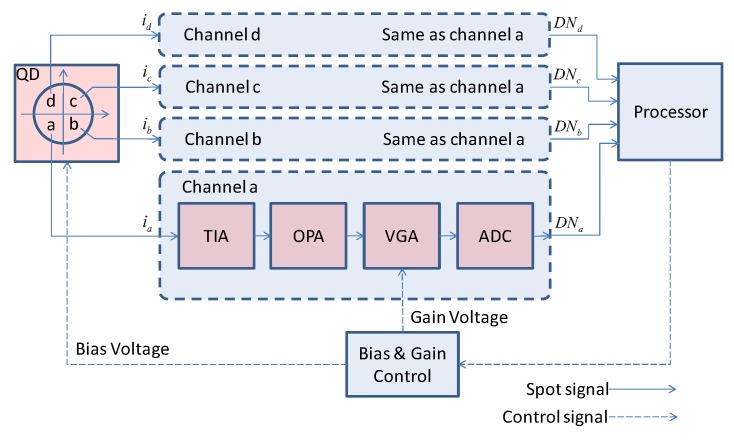
Four-quadrant PIN detector and post-processing circuit schematic.

The four-quadrant PIN detector and post-processing circuit are shown in Figure 3.

The current generated by each quadrant of the four-quadrant PIN detectors enters the channels of the signal processing circuit. Each channel converts the analog current signal into a digital voltage signal for the processor to calculate the displacement of the target by the sum and difference algorithm. At the same time, the processor controls the bias voltage of the four-quadrant PIN detector and the gain of the variable gain amplifier according to the value of the digital signal.As a result, the signal amplitude can always remain within the input range of the ADC to avoid saturation. The structure of each channel of the four-quadrant PIN detector and its post-processing chain is the same.The channel consists of a transmission impedance amplifier (TIA), an operational amplifier (OPA), a variable gain amplifier (VGA) and an analog-to-digital converter (ADC).

**Figure 4:**
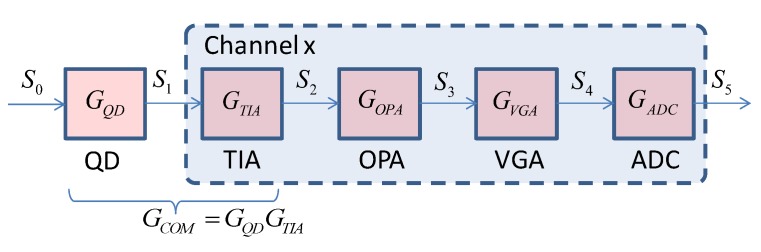
The gain model of QD and its signal processing chain in a channel.

The gain model of QD and its signal processing chain in a channel is shown in Figure 4. $G_{QD}$, $G_{TIA}$, $G_{OPA}$, $G_{VGA}$ and $G_{ADC}$ are the gains of QD, TIA, OPA, VGA and ADC, respectively. The reflected laser $S_{0}$ forms a spot on the surface of QD after passing through the optics. The QD converts the optical power signal $S_{0}$ into a current signal $S_{1}$. The TIA converts $S_{1}$ into a voltage signal $S_{2}$. The secondary amplifier OPA amplifies $S_{2}$ and generates $S_{3}$. The $S_{3}$ is conditioned by VGA to a certain amplitude within the input range of the ADC. The ADC converts the signal $S_{4}$ to a digital signal $S_{5}$ for the processor.

As can be seen from Figure 4, the output of ADC is:

(8) $$S_{5}=G_{QD}G_{TIA}G_{OPA}G_{VGA}G_{ADC}S_{0}$$

The signal-to-noise ratio of the laser semi-active guidance system is:

(9) $$SNR=20\log_{10}\frac{u_{s}}{u_{N\_TOTAL(RTO)}}$$

where $u_{s}$ is the received digital signal derived from the reflected laserradiation from the illuminated object. The $u_{N\_TOTAL(RTO)}$ is the RMS value of system noise measured at the output of the ADC. It is known by Equation (8) that:

(10) $$u_{s}=P_{s}G_{QD}G_{TIA}G_{OPA}G_{VGA}G_{ADC}$$

The $u_{N\_TOTAL(RTO)}$ is contributed by each node which composed the entire signal chain. The noise of QD is a combination of shot noise and thermal noise. The shot noise is the main contributor to the QD noise when a bias voltage is applied to the QD [19]. The noise of QD is given by:

(11) $$i_{N\_QD}=\sqrt{2q\left(i_{s}+i_{d}+i_{B}\right)BW}$$

where $i_{s}$ is the photocurrent generated by $P_{S}$, $i_{B}$ is the photocurrent generated by $P_{B}$, $i_{D}$ is the dart current of the QD, q is electron charge and BW is the noise bandwidth.

The noise of TIA referenced to the input is as follows [20]:

(12) $$i_{N\_TIA(RTI)}=\sqrt{\left(I_{n\_TIA}^{2}+{\left(2\pi fV_{n\_TIA}C_{in}\right)}^{2}+4kT/R_{G\_TIA}\right)BW}$$

where $I_{n\_TIA}$ is the TIA current noise, $V_{n\_TIA}$ is the TIA voltage noise, $C_{in}$ is the total input capacitance seen by the TIA, f is the frequency, k is Boltzmann’s constant, T is temperature in Kelvin, and $R_{G\_TIA}$ is the feedback resistance of the TIA.

The noise of OPA is given by:

(13) $$u_{N\_OPA(RTI)}=\sqrt{\left(V_{n\_OPA}^{2}+4kTR_{3}+4kTR_{1}{\left(\frac{R_{2}}{R_{1}+R_{2}}\right)}^{2}+I_{n+\_OPA}^{2}R_{3}^{2}+I_{n-\_OPA}^{2}R_{3}^{2}{\left(\frac{R_{1}R_{2}}{R_{1}+R_{2}}\right)}^{2}+4kTR_{2}{\left(\frac{R_{2}}{R_{1}+R_{2}}\right)}^{2}\right)BW}$$

where $V_{n\_OPA}$ is the OPA voltage noise, $I_{n+\_OPA}$ is the non-inverting input current noise of OPA, $I_{n-\_OPA}$ is the inverting input current noise of OPA, $R_{1}$ and $R_{2}$ compose the feedback network, and $R_{3}$ is the source impendence of the non-inverting input of OPA.

The noise generated by VGA and ADC will be attenuated by a factor of $G_{TIA}G_{OPA}$ when it is referenced to the input of TIA because the amplification is mainly achieved by TIA and OPA. By selecting a VGA and ADC withlow noise that is equivalent to the noise of TIA, the main contributors of noise in the analog signal process chain are QD, TIA and OPA.

The total noise reference to the output of ADC is as follows:

(14) $$u_{N\_TOTAL(RTO)}=\sqrt{\left(\left(i_{N\_QD}^{2}+i_{N\_TIA(RTI)}^{2}\right)R_{G\_TIA}^{2}+u_{N\_OPA(RTI)}^{2}\right)G_{OPA}^{2}G_{VGA}^{2}G_{ADC}^{2}+u_{N\_VGA(RTI)}^{2}G_{VGA}^{2}G_{ADC}^{2}+u_{N\_ADC(RTI)}^{2}G_{ADC}^{2}}$$

Combing Equations (10)–(14) with Equation (9) we can obtain the SNR as follows:

(15) $$SNR=20\log_{10}\frac{\frac{D^{2}}{4R_{M}^{2}}P_{L}\rho_{T}T_{R}T_{F}e^{-\sigma \left(R_{M}+R_{L}\right)}\cos\theta G_{QD}G_{TIA}G_{OPA}G_{VGA}G_{ADC}}{u_{N\_TOTAL(RTO)}}$$

The distance between the target and the receiving optics $R_{M}$ change from far to near until the missile hits the target during the process of guidance. $R_{M\_MAX}$ is the maximum value of $R_{M}$ which characterizes how far the semi-active laser guidance system can detect targets. The $u_{N\_TOTAL(RTO)\_PP}$ is used to represent the peak-to-peak value of $u_{N\_TOTAL(RTO)}$. For Gaussian noise and a given value of RMS noise, the ratio of $u_{N\_TOTAL(RTO)\_PP}$ to $u_{N\_TOTAL(RTO)}$ is set to 6.6 to obtain the 99.9% of the time noise will not exceed the nominal peak-to-peak value. The ratio of $u_{s}$ to $u_{N\_TOTAL(RTO)\_PP}$ is at least 2 in order to detect the target. Therefore, it is required that the minimum value of the SNR of the receiving system is 22.4 dB in order to detect the target. Meanwhile, the value of $R_{M}$ is $R_{M\_MAX}$. When the ambient temperature changes drastically, $R_{M\_MAX}$ will change accordingly and deviate from the desiredvalue of the design point. It can be seen from Equation (15) that $P_{L}$, $\rho_{T}$, $\rho_{B}$, σ and $E_{\lambda }$ are not changed under the same illumination conditions and target characteristics. D, $T_{R}$ and $T_{F}$ can change a little with temperature by selecting the appropriate material and optimizing the optical design. Usually the characteristics of electronic devices change greatlywith temperature. Therefore, the main factors affecting $R_{M\_MAX}$ are $G_{QD}$, $G_{TIA}$, $G_{OPA}$, $G_{VGA}$, $G_{ADC}$ and $u_{N\_TOTAL(RTO)}$, according to Equation (15), and they will be analyzed in following sections.

## 3. Temperature Characteristics of Four-Quadrant PIN Detector and Its Post-Processing Chain

In order to obtain the temperature characteristic model of the four-quadrant PIN detector and its post-processing circuit, it is necessary to measure the gain-temperature characteristics of each node in the signal-processing chain. This article measures all the nodes of four channels simultaneously to shorten the time cost of the experiment. Since the QD output is a current signal, it is not easy to measure directly. We combine QD and TIA into one unit and measure the voltage signal $S_{2}$ of the TIA output. The $G_{COM}$ can be calculated by:

(16) $$G_{COM}=S_{2}/S_{0}=G_{QD}G_{TIA}$$

The unit of $G_{COM}$ is V/W. Similarly, the $G_{OPA}$, $G_{VGA}$ and $G_{ADC}$ can be calculated by measuring $S_{3}$, $S_{4}$ and $S_{5}$.

(17) $$G_{OPA}=S_{3}/S_{2}$$

(18) $$G_{VGA}=S_{4}/S_{3}$$

(19) $$G_{ADC}=S_{5}/S_{4}$$

The ambient temperature stress is applied to the system under test by a dynamic climate chamber.

**Figure 5:**
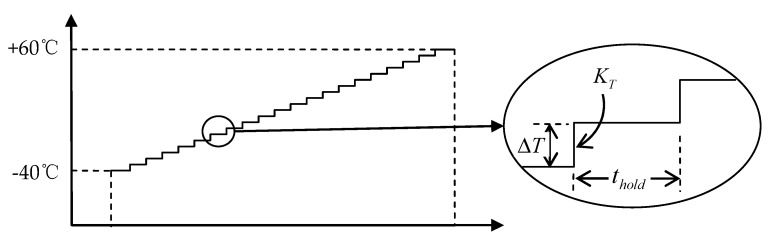
The setting curve of the dynamic climate chamber.

The temperature of the chamber is set to change from −40 °C to +60 °C by a step of ΔT. The four-quadrant PIN detector and the post-processing circuit are kept for $t_{hold}$ at a certain ambient temperature in order to ensure that the temperature of the device under test(DUT) is stable. The slope of temperature change $K_{T}$ is set to 5 °C/min to avoid the thermal shock to the DUT. The setting curve of the dynamic climate chamber is shown in Figure 5, where ΔT is 5 °C and $t_{hold}$ is 20 min.

**Figure 6:**
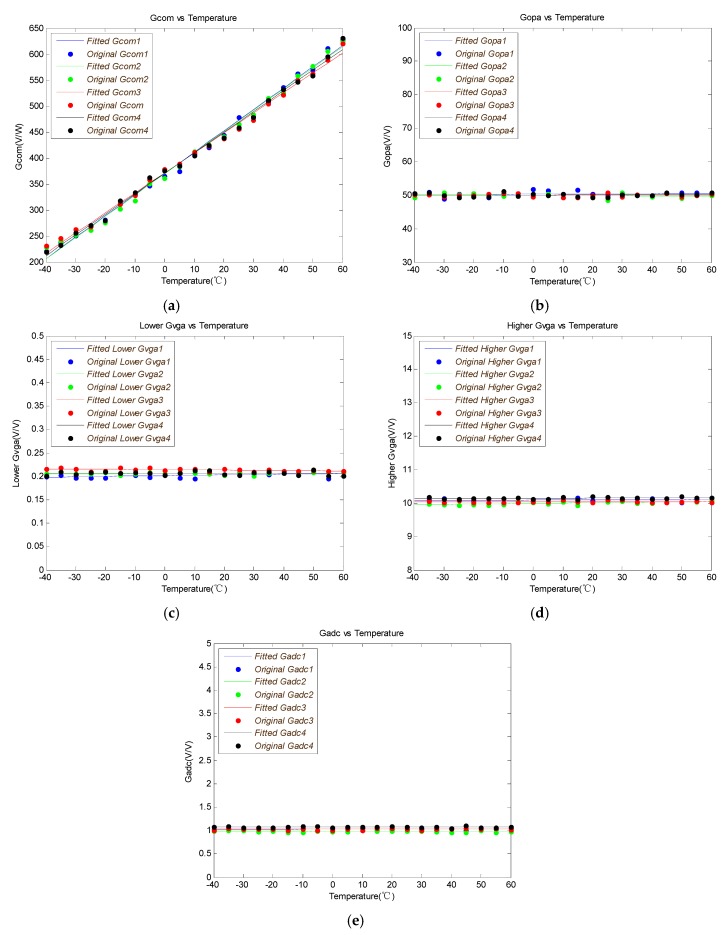
(**a**) $G_{COM}$ versus temperature curve; (**b**) $G_{OPA}$ versus temperature curve; (**c**) $G_{VGA\_MIN}$ versus temperature curve; (**d**) $G_{VGA\_MAX}$ versus temperature curve; (**e**) $G_{ADC}$ versus temperature curve.

The gains of different nodes in the four channels versus temperature curves are measured as shown in Figure 6.

**Figure 7:**
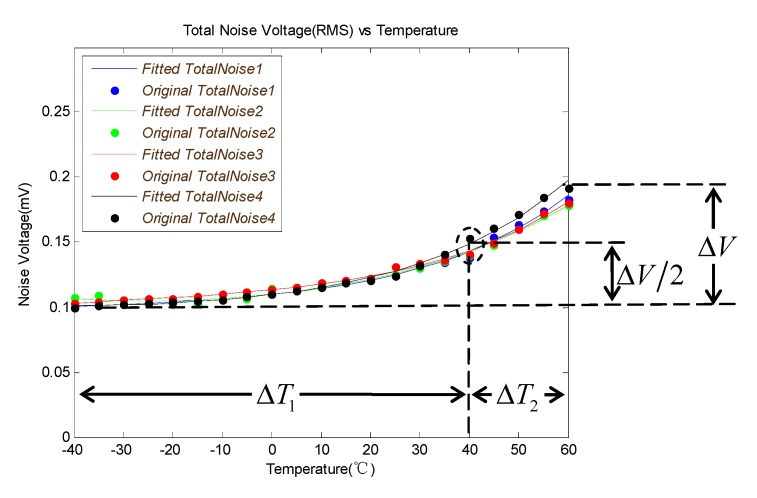
$u_{N\_TOTAL(RTO)}$ versus temperature curve.

The $u_{N\_TOTAL(RTO)}$ versus temperature measured at the output of ADC is shown in Figure 7. The total increment of noise voltage is ΔV when the temperature changes from −40 to 60 °C. And the noise voltage grows ΔV/2 when the temperature changes from −40 to 40 °C ($\Delta T_{1}$). The noise will increase faster when the temperature is higher than 40 °C. It is only about $\Delta T_{2}$ temperature variation which equals a quarter of $\Delta T_{1}$, which can cause another ΔV/2 noise voltage increment when the temperature is higher than 40 °C.

The $G_{COM}$, $G_{OPA}$, $G_{VGA}$,$G_{ADC}$ and $u_{N\_TOTAL(RTO)}$ of four channels are fitted respectively. Since the VGA has the gain, which ranges of from 0.2 to 10, the minimum gain of VGA $G_{VGA\_MIN}$ and the maximum gain of VGA $G_{VGA\_MAX}$ are separately measured and fitted. The parameters of fitted curve for each node of four channels are listed in Table 1.

(20) $$G_{COM\_x}=K1_{COM\_x}t+K2_{COM\_x}$$

(21) $$G_{OPA\_x}=K1_{OPA\_x}t+K2_{OPA\_x}$$

(22) $$G_{VGA\_MIN\_x}=K1_{VGA\_MIN\_x}t+K2_{VGA\_MIN\_x}$$

(23) $$G_{VGA\_MAX\_x}=K1_{VGA\_MAX\_x}t+K2_{VGA\_MAX\_x}$$

(24) $$G_{ADC\_x}=K1_{ADC\_x}t+K2_{ADC\_x}$$

(25) $$u_{N}=K4_{u_{N}\_x}e^{K1_{u_{N}\_x}{\left(t+40\right)}^{3}+K2_{u_{N}\_x}{\left(t+40\right)}^{2}+K3_{u_{N}\_x}(t+40)}$$

The difference characteristics between channels are analyzed first. Theoretically, the gain model is the same for each of the four channels, so the gain differences between the four channels are mainly caused by device processes and materials.

The mean of $K1_{COM\_x}$(x = 1, 2, 3, 4) and the mean of $K2_{COM\_x}$(x = 1, 2, 3, 4) are set to the coefficients of the new fitted curve. The $G_{COM}$ is:

(26) $$G_{COM}=3.998t+370.2$$

It can be concluded that the difference in gain between channels can be negligible by analyzing the coefficient of $G_{OPA}$, $G_{VGA}$, $G_{ADC}$ and $u_{N\_TOTAL(RTO)}$. The new fitted curvesare as follows:

(27) $$G_{OPA}=9.144e^{-5}t+50.09$$

(28) $$G_{VGA\_MIN}=9.197e^{-6}t+0.207$$

(29) $$G_{VGA\_MAX}=4.228e^{-4}t+10.066$$

(30) $$G_{ADC}=-5.473e^{-6}t+1.025$$

(31) $$u_{N}=0.1052e^{1.451e^{-6}{\left(t+40\right)}^{3}-8.84e^{-5}{\left(t+40\right)}^{2}+0.0031(t+40)}$$

## 4. Temperature Compensation for Four-Quadrant PIN Detector and Post-Processing Circuit

The temperature variation of the gain of each node in the signal-processing chain is analyzed in this part. It can be seen from Equations (27)–(30) that the coefficient of the primary term is much smaller than 1, so that the change of $G_{OPA}$, $G_{VGA}$ and $G_{ADC}$ with temperature is not significant. From Equation (26), the primary coefficient is 3.988, so $G_{COM}$ is highly variable with temperature. Therefore, temperature compensation is required for $G_{COM}$.

**Figure 8:**
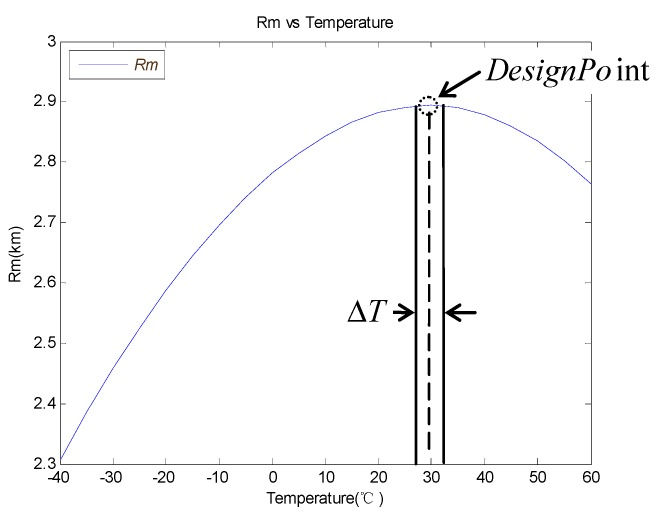
$R_{M\_MAX}$ versus temperature curve.

There is a limit to improving the $R_{M\_MAX}$ by increasing temperature because the $G_{COM}$ and $u_{N\_TOTAL(RTO)}$ are both positively correlated with the temperature. The $R_{M\_MAX}$ changes with temperature are shown in Figure 8, according to Equations (15), (26) and (31). It is obvious that the $R_{M\_MAX}$ increase when the temperature changes from −40 °C to 30 °C and gets to its max value at about 30 °C. When the temperature is higher than 30 °C, the $R_{M\_MAX}$ will decrease because the $u_{N\_TOTAL(RTO)}$ increases faster than the $G_{COM}$. It is necessary to raise the temperature to increase the value of $R_{M\_MAX}$ when the temperature is lower than 30 °C. The $G_{COM}$ is compensated to ensure that it remains above a certain level when the ambient temperature below the design point temperature. In this paper, the system temperature of the design point is selected at 30 °C.

The ideal way to reduce the temperature drift is to control the ambient temperature. However, the ambient temperature control can lead to rapid increases in volume and cost which should be controlled strictly for the semi-active laser guidance system. Therefore, this paper uses local temperature control to compensate for the temperature characteristic of $G_{COM}$ in the system.

**Figure 9:**
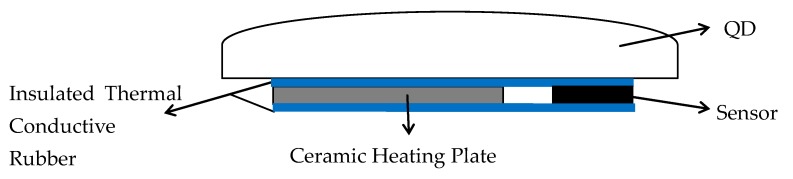
The layout of heater and sensor of temperature control for QD.

The components used for compensation are ceramic heaters and temperature sensors, which are arranged as shown in Figure 9. The QD is placed on the ceramic heating plate. The Pt100 is used to measure the temperature of the QD as a sensor. The insulated thermal conductive rubber is inserted between the QD and the ceramic electric heater to improve the thermal conductivity.

**Figure 10:**
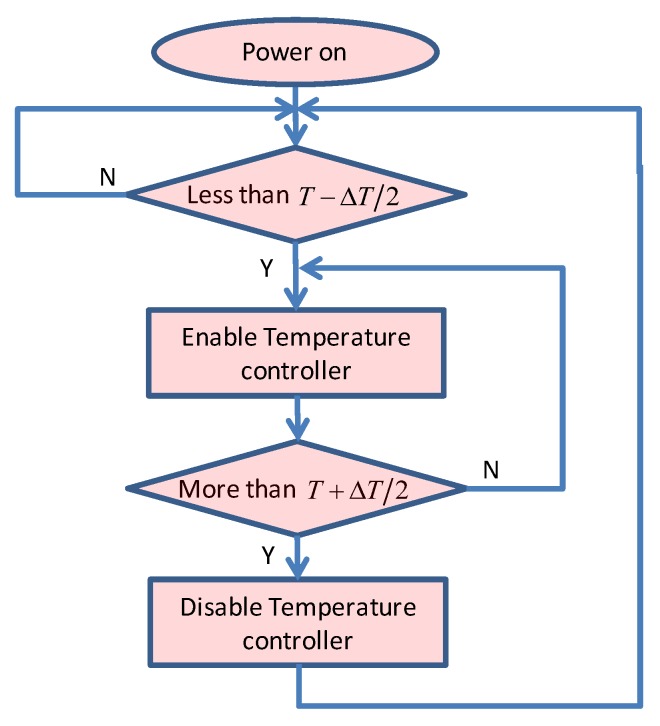
The process of temperature control for QD.

The target temperature is set to 30 °C. A temperature tolerance ΔT is necessary to avoid the temperature controller switching frequently between on and off when the temperature is near to the design point. In this paper, the ΔT is set to 3 °C and the $R_{M\_MAX}$ changes little when the temperature is in the tolerance of the design point. The control flow chart is shown in Figure 10. When the temperature measured by the sensor is less than T−ΔT/2 °C, the temperature controller is enabled to heat the QD until the temperature of QD is more than T+ΔT/2.

## 5. Verification for the System under Compensation

**Figure 11:**
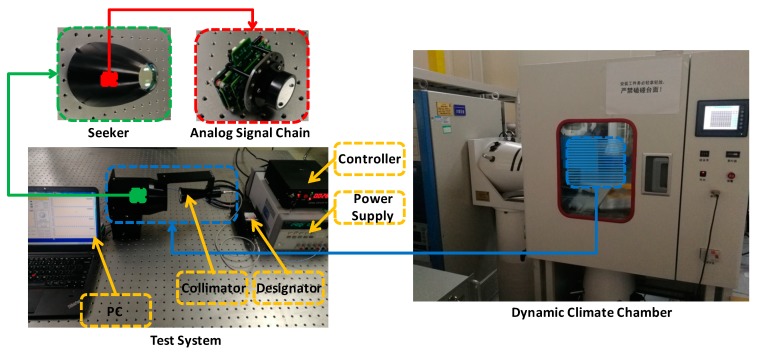
The test system and dynamic climate chamber.

The test system and dynamic climate chamber are shown in Figure 11. The test system is composed of PC, seeker, designator, collimator, controller, and power supply. The seeker with analog signal chain and optics is powered by a DC power supply. The designator can send the laser that is coupled by an optical fiber to the collimator. The frequency and width of the laser emitted from the designator are controlled by the controller. The test data is collected by PC for post process. The seeker and collimator will be put into the dynamic climate chamber when applying environmental stress to the seeker and verifying the effect of compensation.

**Figure 12:**
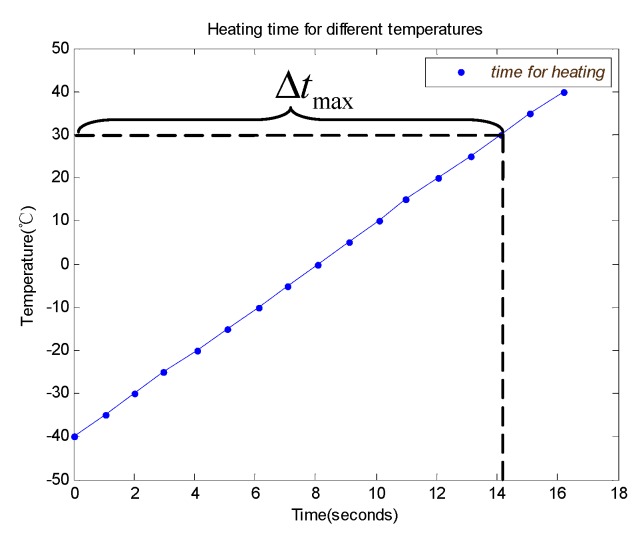
The heating time for different ambient temperatures.

The ambient temperature stress, shown in Figure 5, is applied to the system with compensation. The heating time during different ambient temperatures is obtained, as shown in Figure 12.

The heating time depends on the heating power and heat capacity of the component being heated and the ambient temperature. The system needs a long time for heating when it starts up at a low ambient temperature. The maximum heating time, $\Delta t_{\max}$, is 14.11 s when the system is heated from −40 °C to 30 °C. The average temperature rise rate is about 5 °C per second.

**Figure 13:**
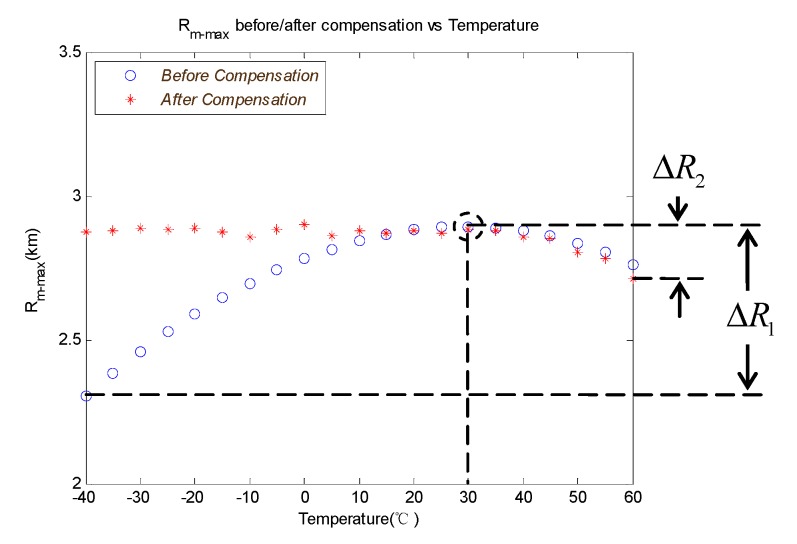
Comparison of the $R_{M\_MAX}$ under different ambient temperatures before and after compensation.

As the typical working conditions given in Table 2 show, the $R_{M\_MAX}$ that is not compensated changes with the ambient temperature, as shown by the blue curve in Figure 13. The $R_{M\_MAX}$ that is compensated changes with the ambient temperature, as shown by the red curve in Figure 13.

It can be seen from Figure 13 that the ratio of maximum variation of detection range $\Delta R_{1}$ to the desire value of design point is 20.1% when the ambient temperature changes from −40 °C to 60 °C without temperature compensation. After the temperature compensation, the ratio of $\Delta R_{2}$ to the desire value of design point can be optimized to 6.9%.

## 6. Conclusions

In this paper, the mathematical model of the detection range of the semi-active laser guidance system is established. The temperature characteristic of each node of the analog signal chain is measured and the main factors with dramatic changes are screened. The relationship between the $R_{M\_MAX}$ and the temperature is deduced and the design point is selected based on it. Compensation is applied to the system when the ambient temperature changes from−40 °C to 30°C.The ratio of the maximum variation of the detection range to the design point is 6.9% and 20.1% with compensation and without compensation, respectively. It can be concluded that this temperature compensation method can improve the detection range of semi-active laser guidance system in a wide temperature range. Furthermore, the ratio of the maximum variation of the detection range to the design point can be optimized to nearly zero when a thermoelectric cooler (TEC) is applied when the ambient temperature is higher than the design point. However, the cooling efficiency of TEC is relatively low and the typical cooling efficiency can reach 50%. It needs a large amount of energy from a power supply and a long time to cool the detector to 30 °C when the ambient temperature is 60 °C.

## Figures and Tables

**Table 1:** The parameters of fitted curve for each node of four channels.

	x = 1	x = 2	x = 3	x = 4	Mean Value	Standard Deviation
$K1_{COM\_x}$	4.095	4.084	3.850	3.964	3.998	0.1151
$K2_{COM\_x}$	370.3	369.1	371.0	370.4	370.2	0.7951
$K1_{OPA\_x}$	0.004171	−0.004646	−0.0001631	0.001004	9.144 × 10^−5^	0.003651
$K2_{OPA\_x}$	50.29	49.99	50.02	50.05	50.09	0.1360
$K1_{VGA\_MIN\_x}$	8.059 × 10^−5^	1.045 × 10^−5^	−4.557 × 10^−5^	−8.678 × 10^−6^	9.198 × 10^−6^	5.297 × 10^−5^
$K2_{VGA\_MIN\_x}$	0.2016	0.2063	0.2144	0.2068	0.2073	0.005267
$K1_{VGA\_MAX\_x}$	0.0003600	0.0009979	9.079 × 10^−5^	0.0002427	0.0004228	0.0003989
$K2_{VGA\_MAX\_x}$	10.09	9.991	10.04	10.14	10.07	0.06547
$K1_{ADC\_x}$	1.968 × 10^−6^	−0.0001066	0.0001174	−3.459 × 10^−5^	−5.473 × 10^−6^	9.350 × 10^−5^
$K2_{ADC\_x}$	1.025	0.9789	1.025	1.071	1.025	0.03765
$K1_{u_{N}\_x}$	5.860 × 10^−7^	3.918 × 10^−8^	4.747 × 10^−7^	4.402 × 10^−7^	3.850 × 10^−7^	2.388 × 10^−7^
$K2_{u_{N}\_x}$	−1.792 × 10^−5^	6.799 × 10^−5^	−1.242 × 10^−5^	1.5706 × 10^−5^	1.335×10^−5^	3.930 × 10^−5^
$K3_{u_{N}\_x}$	0.0021	−0.002	0.0021	7.658 × 10^−4^	7.496 × 10^−4^	0.0020
$K4_{u_{N}\_x}$	0.100	0.1065	0.1031	0.1009	0.1027	0.0029

**Table 2:** Typical working conditions of semi-active laser guidance system.

Parameter	Value	Parameter	Value	Parameter	Value	Parameter	Value
$R_{L}$	7 km	$T_{R}$	0.8	$E_{T}$	80 mJ	$\Delta_{\lambda }$	40 nm
τ	20 ns	$T_{F}$	0.8	$\theta_{L}$	65°	θ	65°
$\beta_{T}$	1 mrad	D	25 mm	$\rho_{T}$	0.2	$\rho_{B}$	0.2
